# Measurement of Unmanned Aerial Vehicle Attitude Angles Based on a Single Captured Image

**DOI:** 10.3390/s18082655

**Published:** 2018-08-13

**Authors:** Jin Zhang, Lijun Ren, Huaxia Deng, Mengchao Ma, Xiang Zhong, Pengcheng Wen

**Affiliations:** 1 School of Instrument Science and Opto-electronics Engineering, Hefei University of Technology, No. 193 Tunxi Road, Hefei 230009, China; zhangjin@hfut.edu.cn (J.Z.); hfutrlj@mail.hfut.edu.cn (L.R.); mmchao@hfut.edu.cn (M.M.); 2 AVIC Xi’an Aeronautics Computing Technique Research Institute, Xi’an 710000, China; wpcheng@avic.com

**Keywords:** monocular vision, optical measurement, attitude angle, coordinate transformation, coded target, UAV

## Abstract

The limited load capacity and power resources of small-scale fixed-wing drones mean that it is difficult to employ internal high-precision inertial navigation devices to assist with the landing procedure. As an alternative, this paper proposes an attitude measurement system based on a monocular camera. The attitude angles are obtained from a single captured image containing five coded landmark points using the radial constraint method and three-dimensional coordinate transformations. The landing procedure is simulated for pitch angles from −15${}^{\circ }$ to −40${}^{\circ }$, roll angles from −15${}^{\circ }$ to +15${}^{\circ }$ and yaw angles from −15${}^{\circ }$ to +15${}^{\circ }$. For roll and pitch angles of approximately 0${}^{\circ }$ and −25${}^{\circ }$, respectively, the accuracy of the method reaches 0.01${}^{\circ }$ and 0.04${}^{\circ }$. This UAV attitude measurement system obtains an attitude angle by a single captured image, which has great potential for assisting with the landing of small-scale fixed-wing UAVs.

## 1. Introduction

High-precision measurement of the attitude angles of an unmanned aerial vehicle (UAV), i.e., a drone, is of critical importance for the landing process. In general, the attitude angles of a UAV are measured by an internal device [1,2], such as a gyroscope [3,4,5] or a GPS angle measurement system [6,7,8]. However, for small-scale fixed-wing drones, limitations on load capacity and power supply make the use of internal high-precision gyroscopes impracticable, and instead an approach based on external attitude measurement must be adopted. External attitude measurement methods determine a drone’s attitude angles by using external measuring devices such as theodolites or cameras. Such methods are divided into two types according to where the measurement device is installed: a visual sensor can either be mounted on the drone or located at some position away from it [9]. Installation of the sensor away from the drone provides high accuracy, but using the device is inconvenient for navigating the drone. Therefore, to assist in the landing of small-scale drones, methods employing drone-mounted visual sensors are most commonly used [10,11,12]. With the development of visual sensor technology, visual sensors could be implemented even to the smaller drones in the near future, and the computational capacity together with the camera quality will be on a better technological level.

Most methods based on visual techniques to obtain the attitude angles of a UAV require multiple images with a fixed-focus lens. Eynard et al. [13] presented a hybrid stereo system for vision-based navigation composed of a fisheye and a perspective camera. Rawashdeh et al. [14] and Ettinger et al. [15] obtained the attitude angles by detecting changes in the horizon between two adjacent images from a single fisheye camera. Tian and Huang [16], Caballero et al. [17], and Cesetti et al. [18] matched features of sequential images from a single camera to obtain attitude angles by capturing natural landmarks on the ground. Li et al. [19] obtained the pose with the uncalibrated multi-view images and the intrinsic camera parameters. Dong et al. [20] matched dual-viewpoint images of a corner target to find the attitude angles. All of these methods obtain the drone’s attitude angles through the use of multiple images. However, fast flying speeds and constant changes in attitude angles lead to an attitude angle delay, with the result that the attitude angles acquired using these methods are not the current angles. In addition, the accuracy of most of these methods based on monocular cameras is dependent on the focal length of the lens employed. For example, with the method proposed by Tian and Huang [16], the accuracy is proportional to focal length. Eberli et al. [21] imaged two concentric circles with a fixed-focus lens and determined the attitude angles from the deformation of these circles. Also using a fixed focal length, Li [22] measured the angle of inclination of a marker line, from which he obtained the attitude angles. Soni and Sridhar [23] proposed a pattern recognition method for landmark recognition and attitude angle estimation, using a fixed-focus lens. In the method proposed by Gui et al. [24], the centers of four infrared lamps acting as ground targets were detected and tracked using a radial constraint method, with the accuracy again being dependent on the focal length of the lens. During landing of a UAV, as the distance to a cooperative target decreases, the view size of the target changes. Thus, in addition to attitude angle delay, most of the existing methods for assisting with the landing of a small fixed-wing drone also suffer from the problem of varying view size of cooperative targets.

In contrast to existing methods, our method determines the UAV attitude angles from just a single captured image containing five coded landmark points using a zoom system. Not only does this method reduce angle delay, but the use of a zoom system greatly improves the view size of the cooperative target. The remainder of this paper is organized as follows: Section 2 explains the principle of the scheme for obtaining the attitude angles, Section 3 describes a simulation experiment, Section 4 presents the experimental results and a discussion, and Section 5 gives our conclusions.

## 2. Measurement Scheme

The UAV attitude angles are obtained from a single captured image containing five coded landmark points using the radial constraint method together with a three-dimensional coordinate transformation.

**Figure 1:**
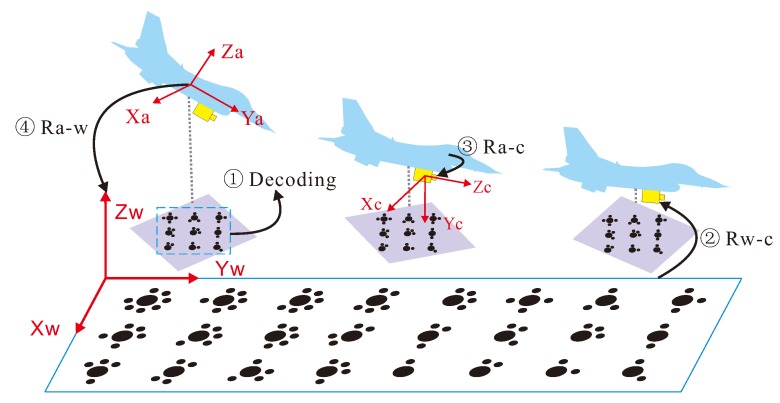
Solution model for obtaining the attitude angles for UAV landing.

The solution procedure is divided into four steps, as shown in Figure 1. The first step is to decode and obtain the relationship between the coordinates of the code mark point in the image coordinate system and those in the world coordinate system. In the second step, the principal point $\left(u_{0},v_{0}\right)$ is calibrated, and the rotation matrix $R_{w-c}$ between the world coordinate system and the camera coordinate system is obtained by the radial constraint method. The third step determines the rotation matrix $R_{a-c}$ between the UAV coordinate system and the camera coordinate system according to the rules for converting between three-dimensional coordinate systems. In the fourth step, the rotation matrix $R_{a-w}$ between the UAV coordinate system and the world coordinate system is obtained from the matrices found in the second and third steps, and is then used to calculate the UAV attitude angles.

### 2.1. Coded Target Decoding

**Figure 2:**
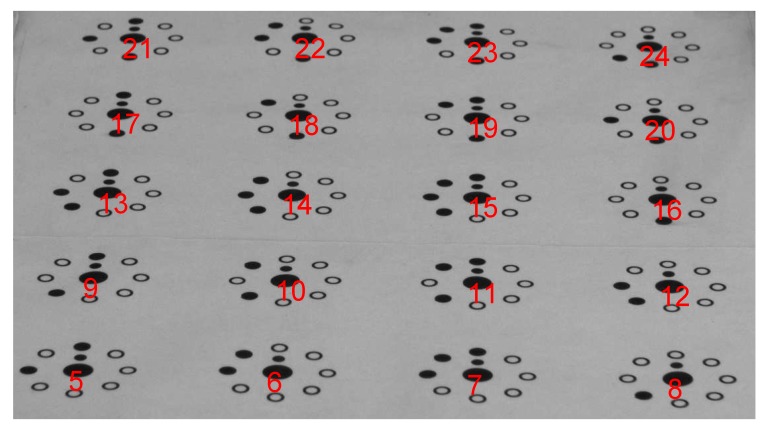
Code target decoding diagram.

The marking point used in this paper is a coded marking point with a starting point. The coding method is binary coding. The closest point to the initial point is the starting point of the code. The coding is counterclockwise, and each circle at the outermost periphery represents one bit. Figure 2 shows one of the captured encoded target maps. The relationship between the coordinates in the image coordinate system and the corresponding coordinates in the world coordinate system is obtained through a decoding calculation.

From the 20 successfully decoded points in Figure 2, five are selected to solve for the attitude angles, as shown in Table 1. $\left(X_{w},Y_{w}\right)$ denote coordinates in the world coordinate system, and (u,v) denote those in the image coordinate system.

### 2.2. Solution for the Rotation Matrix between the World and Camera Coordinate Systems

**Figure 3:**
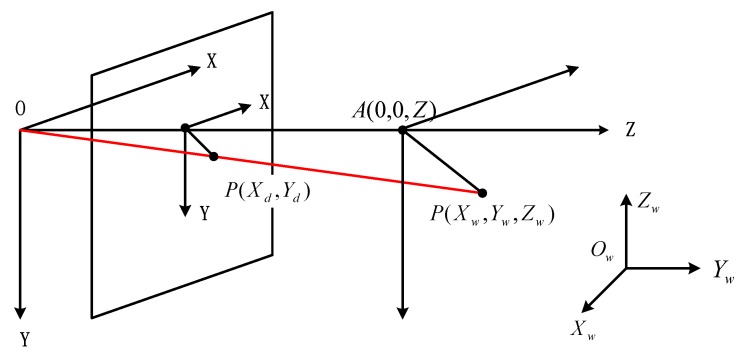
Schematic diagram showing the principle of the radial restraint method.

The radial constraint method is used to find the rotation matrix between the camera coordinate system and the world coordinate system. According to the ideal camera imaging model, as shown in Figure 3, $P(X_{w},Y_{w},Z_{w})$ is a point in the world coordinate system, while the corresponding point in the camera plane coordinate system is $P(X_{d},Y_{d})$. It should be noted that none of the equations derived using the radial constraint method involve the effective focal length *f*. It is important to choose the appropriate focal length based on the distance between the camera and the cooperative target. This is the main reason why in this paper we choose the radial constraint method to obtain the rotation matrix $R_{w-c}$.

In practical applications, the target is planar, so $Z_{w}=0$. Thus, the relevant formula is

(1) $$\frac{x}{y}=\frac{X_{d}}{Y_{d}}=\frac{r_{1}X_{w}+r_{2}Y_{w}+T_{x}}{r_{4}X_{w}+r_{5}Y_{w}+T_{y}}.$$

Here, $\left(X_{d},Y_{d}\right)$ denotes the actual image coordinates and $\left(X_{w},Y_{w}\right)$ denotes the world coordinates. Finally, the unit orthogonality of $R_{w-c}$ is used to determine $r_{3}$, $r_{6}$, $r_{7}$, $r_{8}$, and $r_{9}$ [25]. The rotation matrix $R_{w-c}$ from the world coordinate system to the camera coordinate system is thereby obtained.

### 2.3. Solution for the Rotation Matrix between the UAV and Camera Coordinate Systems

The camera is mounted on the drone at a known position, and so the rotation matrix between the UAV coordinate system and the camera coordinate system can be obtained from a three-dimensional coordinate transformation. The *X*, *Y*, and *Z* axes of the UAV coordinate system are rotated through angles λ, θ, and ϕ around the $X_{c}$, $Y_{c}$, and $Z_{c}$ axes, respectively, of the camera coordinate system. The rotation matrices representing rotations through angles λ, θ, and ϕ around the *X*, *Y*, and *Z* axes, respectively, are

(2) $$\begin{array}{cc} R_{x}\left(\lambda \right) & =\left[\begin{array}{ccc} 1 & 0 & 0\\ 0 & \cos\lambda & -\sin\lambda \\ 0 & \sin\lambda & \cos\lambda \end{array}\right], \end{array}$$

(3) $$\begin{array}{cc} R_{y}\left(\theta \right) & =\left[\begin{array}{ccc} \cos\theta & 0 & \sin\theta \\ 0 & 1 & 0\\ -\sin\theta & 0 & \cos\theta \end{array}\right], \end{array}$$

(4) $$\begin{array}{cc} R_{z}\left(\phi \right) & =\left[\begin{array}{ccc} \cos\phi & -\sin\phi & 0\\ \sin\phi & \cos\phi & 0\\ 0 & 0 & 1 \end{array}\right]. \end{array}$$

Therefore, the rotation matrix between the UAV coordinate system and the camera coordinate system is given by the matrix product

(5) $$R_{a-c}=R_{z}\left(\phi \right)R_{y}\left(\theta \right)R_{x}\left(\lambda \right).$$

The relative positions of the UAV and the camera in the simulation experiment are shown in Figure 1. The angles between the *X*, *Y*, and *Z* axes of the UAV coordinate system and the $X_{c}$, $Y_{c}$, and $Z_{c}$ axes of the camera coordinate system are $0^{\circ }$, $90^{\circ }$, and $90^{\circ }$, respectively. According to Formula (5), the rotation matrix between the UAV coordinate system and the camera coordinate system is then given by

(6) $$R_{a-c}=R_{z}\left(\phi \right)R_{y}\left(\theta \right)R_{x}\left(\lambda \right)=\left[\begin{array}{ccc} 0 & -1 & 0\\ 0 & 0 & 1\\ -1 & 0 & 0 \end{array}\right].$$

### 2.4. Solution for the Rotation Matrix between the UAV and World Coordinate Systems

**Figure 4:**
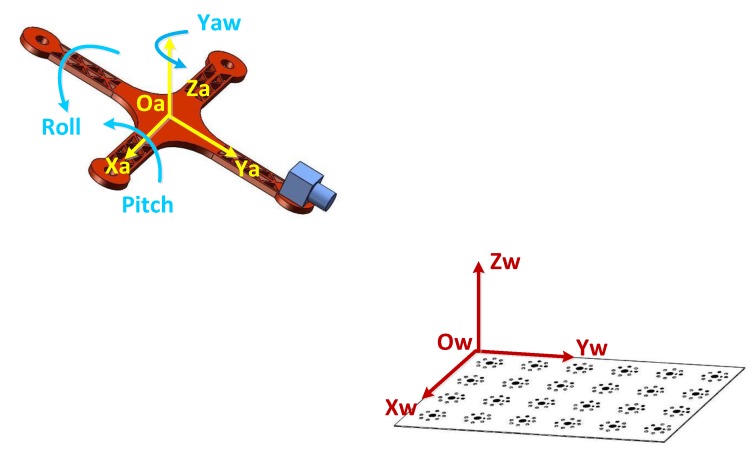
The relationship between the UAV coordinate system and the world coordinate system.

The two steps above have provided the rotation matrix $R_{w-c}$ from the world coordinate system to the camera coordinate system and the rotation matrix $R_{a-c}$ from the UAV coordinate system to the camera coordinate system. Given a point P(X,Y,Z), we denote by $P_{w}\left(X_{w},Y_{w},Z_{w}\right)$, $P_{c}\left(X_{c},Y_{c},Z_{c}\right)$, and $P_{a}\left(X_{a},Y_{a},Z_{a}\right)$ the corresponding points in the world, camera, and UAV coordinate systems, respectively. We denote by $T_{w-c}$ and $T_{a-c}$ the translation matrices between the world coordinate system and the camera coordinate system and between the UAV coordinate system and the camera coordinate system, respectively. Figure 4 shows the relationship between the UAV coordinate system and the world coordinate system.

The coordinates of the point $P_{c}$ in the camera coordinate system can be obtained from the relationship between the camera and world coordinate systems as

(7) $$P_{c}=R_{w-c}P_{w}+T_{w-c}$$

and from the relationship between the UAV and camera coordinate systems as

(8) $$P_{c}=R_{a-c}P_{a}+T_{a-c}.$$

Equating these two expressions for $P_{c}$ gives

(9) $$R_{w-c}P_{w}+T_{w-c}=R_{a-c}P_{a}+T_{a-c},$$

from which we have

(10) $$P_{w}=R_{w-c}^{-1}R_{a-c}P_{a}+R_{w-c}^{-1}\left(T_{a-c}-T_{w-c}\right).$$

The rotation matrix between the UAV coordinate system and the world coordinate system is given by

(11) $$R_{a-w}=R_{w-c}^{-1}R_{a-c}=\left|\begin{array}{ccc} \cos\theta \cos\phi & \sin\lambda \sin\theta \cos\phi -\cos\lambda \sin\phi & \cos\lambda \sin\theta \cos\phi +\sin\lambda \sin\phi \\ \cos\theta \sin\phi & \sin\lambda \sin\theta \sin\phi +\cos\lambda \cos\phi & \cos\lambda \sin\theta \sin\phi -\sin\lambda \cos\phi \\ -\sin\theta & \sin\lambda \cos\theta & \cos\lambda \cos\theta \end{array}\right|$$

where λ, θ, and ϕ now denote the angles through which the *x*, *x*, and *z* axes of the UAV coordinate system are rotated around the *x*, *y*, and *z* axes, respectively, of the world coordinate system. The attitude angles of UAV are obtained from the following formulas:

(12) $$\left\{\begin{array}{c} \theta_{1}=-\arcsin R_{31}/\pi \times 180,\\ \theta_{2}=\pi -\theta_{1}, \end{array}\right.$$

(13) $$\left\{\begin{array}{c} \lambda_{1}=\mathrm{atan}2\;\left(\frac{R_{32}}{\cos\theta_{1}},\frac{R_{33}}{\cos\theta_{1}}\right)/\pi \times 180,\\ \lambda_{2}=\mathrm{atan}2\;\left(\frac{R_{32}}{\cos\theta_{2}},\frac{R_{33}}{\cos\theta_{2}}\right)/\pi \times 180, \end{array}\right.$$

(14) $$\left\{\begin{array}{c} \phi_{1}=\mathrm{atan}2\;\left(\frac{R_{21}}{\cos\theta_{1}},\frac{R_{11}}{\cos\theta_{1}}\right)/\pi \times 180,\\ \phi_{2}=\mathrm{atan}2\;\left(\frac{R_{21}}{\cos\theta_{2}},\frac{R_{11}}{\cos\theta_{2}}\right)/\pi \times 180. \end{array}\right.$$

According to Formulas (12)–(14), the algorithm proposed in this paper is able to achieve the measurement of three attitude angles (yaw angle, pitch angle and roll angle). The solution of the attitude angle is independent of the focal length. It should be noted that the relative positions of the UAV and the camera in practical applications can be adjusted by pan-tilt. Regardless of whether the drone’s pitch angle is positive or negative, the attitude of the drone can be solved by adjusting the attitude of the camera through the pan/tilt. The rotation matrix between the UAV coordinate system and the camera coordinate system should also be re-acquired correspondingly according to the position relationship.

## 3. Experiment

**Figure 5:**
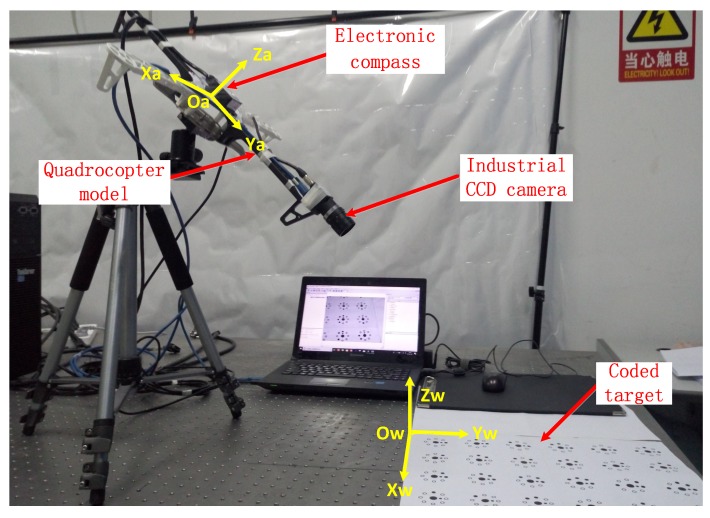
Experimental setup.

The platform for the experiment was constructed with a coded target for landing cooperation, as shown in Figure 5. The quadrocopter model was equipped with an industrial CCD camera and an electronic compass for measuring its attitude angles. The imaging area of the CCD camera was 1292×964 pixels. The sensor size was 1/3 inch and the pixel size was 3.75×3.75 μm. Four lenses with focal lengths of 12, 16, 25, and 35 mm were used. The electronic compass pitch accuracy, roll accuracy, and heading accuracy were $0.15^{\circ }$, $0.15^{\circ }$, and $0.8^{\circ }$, respectively. Capturing of images and electronic compass data acquisition were both under computer control. The electronic compass and camera were fixed on the four-axle aircraft model, with the compass in the middle and the camera in the front. The four-axis aircraft model was fixed on a tripod using a pan–tilt–zoom (PTZ) camera. The target was a planar coding target with an initial point, and the distance between target and camera was in the range 50–300 cm.

Determination of the world coordinate system $O_{w}$–$X_{w}Y_{w}Z_{w}$ was based on the four-axis aircraft model. When the pitch, roll, and yaw angles of the four-axis aircraft model were all equal to $0^{\circ }$, the world coordinate system was taken as the coordinate system of the four-axis aircraft model $O_{a}$–$X_{a}Y_{a}Z_{a}$. The world coordinate system $O_{w}$–$X_{w}Y_{w}Z_{w}$ was not fixed in the horizontal direction. Moving the target in parallel had no effect on the experimental results. After the world coordinate system was determined, the coding target was fixed to the optical experimental platform. It was necessary to ensure that there were at least five coded landmarks, in order to allow measurement of the attitude angles of the four-axis aircraft.

## 4. Results and Discussion

To validate the accuracy of the attitude angle and the effect of focal length on accuracy, the experiment was designed on the basis of the specification “calibration specification for moving pose measurement system”. In the verification experiment, four groups of experiments were performed for simulating the variation of attitude angles during the landing of the drone in which the pitch angle was changed from $-15^{\circ }$ to $-40^{\circ }$, the roll angle from $-15^{\circ }$ to $15^{\circ }$ and the yaw angle from $-15^{\circ }$ to $15^{\circ }$. Furthermore, the dependency between the three angles was also added to the discussion.

In the first group of experiments, during the landing process, the distance between the drone and the cooperative target decreased continuously. In order to identify the influences of focal lengths on the attitude angles measurement, lenses of different focal lengths (12, 16, 25, and 35 mm) were used to photograph the target with the drone in the same pose, with a pitch angle of about $-25^{\circ }$, a roll angle of about $0^{\circ }$ and a yaw angle of about $0^{\circ }$. In the second group of experiments, the yaw angle measurement was identified in the range from $-15^{\circ }$ to $15^{\circ }$ with a pitch angle of about $-30^{\circ }$ and a roll angle of about $0^{\circ }$. The condition of close landing with a yaw angle of about $0^{\circ }$ was also investigated. The roll and pitch angles were changed in increments of $5^{\circ }$ from $-15^{\circ }$ to $15^{\circ }$ and $-15^{\circ }$ to $-40^{\circ }$ in this condition. Similarly, the roll angles and pitch angles were investigated in the third and fourth group, respectively. In the third group, for roll angles of $-15^{\circ }$, $-10^{\circ }$, $-5^{\circ }$, $0^{\circ }$, $5^{\circ }$, $10^{\circ }$, and $15^{\circ }$, the pitch angle was changed in $5^{\circ }$ increments from $-15^{\circ }$ to $-40^{\circ }$ with yaw angle at $0^{\circ }$ unchanged. In the fourth group, for pitch angles of $-15^{\circ }$, $-20^{\circ }$, $-25^{\circ }$, $-30^{\circ }$, $-35^{\circ }$, and $-40^{\circ }$, the roll angle was changed in increments of $5^{\circ }$ from $-15^{\circ }$ to $15^{\circ }$ with yaw angle at $0^{\circ }$ unchanged.

### 4.1. Focal Lengths

During landing of a UAV, as the distance to a cooperative target decreases, the use of a zoom system greatly improves the view size of the cooperative target and further improves the accuracy of the attitude angle measurement. The proposed algorithm for the UAV attitude angle was irrelevant to the focal length as mentioned in the measurement scheme section. Lenses of different focal lengths (12, 16, 25, and 35 mm) were used to photograph the target with the drone in the same pose, with a pitch angle of about $-25^{\circ }$, a roll angle of about $0^{\circ }$ and a yaw angle of about $0^{\circ }$. The verification experiment is as follows.

Table 2 compares the experimental result and ground truth (electronic compass values) for attitude angles at different lens’ focal lengths. The average error in the pitch angle is 0.36${}^{\circ }$, and the minimum error reaches 0.04${}^{\circ }$. Similarly, for roll angles and yaw angles, the average errors are 0.40${}^{\circ }$, 0.38${}^{\circ }$ respectively, and the minimum error of 0.01${}^{\circ }$, 0.04${}^{\circ }$, respectively. The results illustrate that the attitude angles of a UAV can be determined with high accuracy using the proposed method when these angles remain nearly constant during descent and that the accuracy is independent of the focal length of the camera lens. Furthermore, during landing of the drone, the accuracy can be increased by appropriate selection of focal length depending on the distance between the UAV and the cooperative target.

### 4.2. Yaw Angles

Yaw angle is important for UAV control, especially when precise and restricted landing direction and location are required to overcome the cross wind components. At earlier stages of the final approach for instance 200 m out, GPS and altimeters are sufficient given glide path. At 10 m or less to landing pitch, the proposed method has the capability to provide the attitude angles information including pitch, roll and yaw.

**Figure 6:**
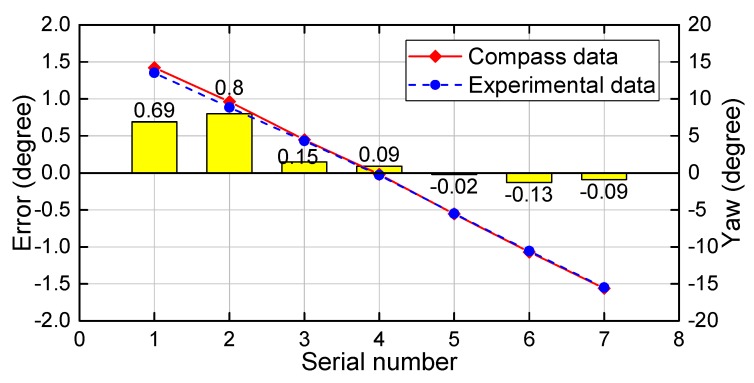
Yaw angle comparison with pitch and roll angles at $-30^{\circ }$ and $0^{\circ }$.

The comparison between electronic compass data and experimental data for the roll angle are presented in Figure 6 at different yaw angles from $-15^{\circ }$ to $+15^{\circ }$. The red line indicates the compass data, and the blue line indicates the experimental data. The yellow histogram shows the error between the experimental data and the compass data. The experimentally determined yaw angles are almost coincident with the actual angles at each measurement point on the graph in which the minimum error in the yaw angle reaches 0.02${}^{\circ }$, the average error reaches 0.28${}^{\circ }$ and the maximum error reaches 0.8${}^{\circ }$ (errors here are absolute). The yaw angle has high accuracy when yaw angles vary around 0${}^{\circ }$, and the error increases with the increases of the yaw angle.

Results in Table 3 compare the yaw angles with the roll angle varying from $-15^{\circ }$ to $+15^{\circ }$, in which the minimum error in the yaw angle reaches 0.05${}^{\circ }$ and the average error reaches 0.43${}^{\circ }$. Similarly, Table 4 compares the yaw angles with the pitch angle varying from $-15^{\circ }$ to $-40^{\circ }$, in which the minimum error in the yaw angle reaches 0.05${}^{\circ }$ and the average error reaches 0.49${}^{\circ }$. The yaw angle achieves high accuracy as roll angles vary around 0${}^{\circ }$. The experimental results show that the proposed method achieves high accuracy in the yaw angle, and the error of yaw angles is less than $1^{\circ }$ with the variation of pitch angles and roll angles.

### 4.3. The Pitch and Roll Angles

During the landing of the drone, the pitch angle of the drone gradually decreases, and the roll is with slight variations (affected by the cross wind). In the verification experiment, roll and pitch angles were changed in increments of $5^{\circ }$ from $-15^{\circ }$ to $15^{\circ }$ and $-15^{\circ }$ to $-40^{\circ }$ with a yaw angle of about $0^{\circ }$ unchanged.

The experimental results are compared with the electronic compass data for six different locations in Table 5. In the following discussion, the electronic compass data are taken as giving the true attitude angles for the quadrocopter model. The attitude angles for these six positions were all measured when the roll angle was close to $0^{\circ }$, which means that the quadrocopter model did not roll during the simulated landing. The pitch angle of the quadrocopter model decreased from $-15^{\circ }$ to $-40^{\circ }$. When the roll and pitch angles were approximately $0^{\circ }$ and $-25^{\circ }$, respectively, the accuracies of the experimental results for these angles reached $0.01^{\circ }$ and $0.04^{\circ }$, thus indicating that the method proposed in this paper achieves high accuracy when the roll angle changes little during descent of the drone.

**Figure 7:**
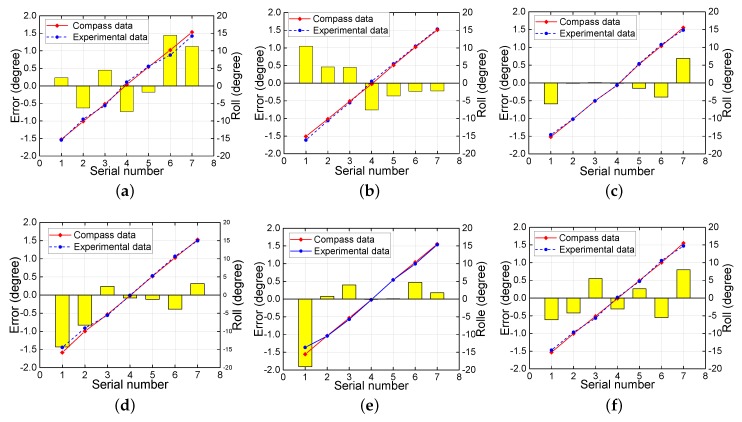
Roll angle comparison at pitch angles of (**a**) $-15^{\circ }$; (**b**) $-20^{\circ }$; (**c**) $-25^{\circ }$; (**d**) $-30^{\circ }$; (**e**) $-35^{\circ }$; and (**f**) $-40^{\circ }$.

**Figure 8:**
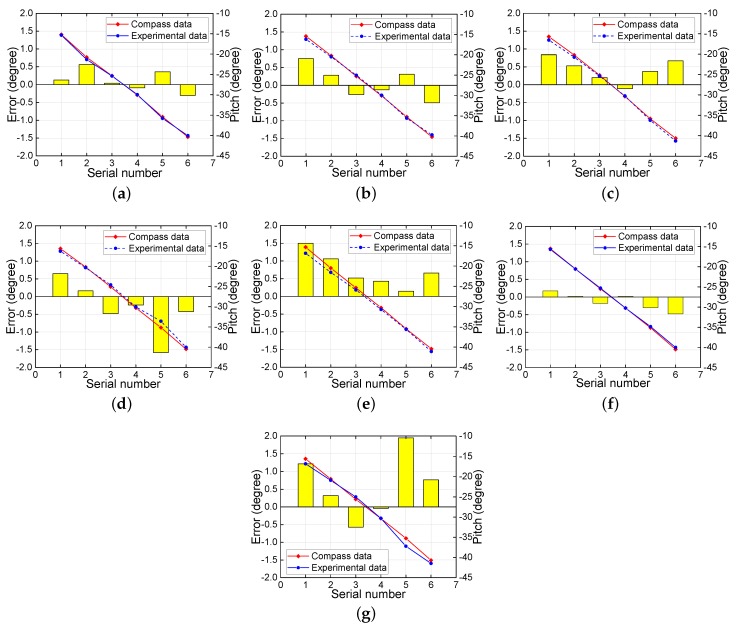
Pitch angle comparison at roll angles of (**a**) $0^{\circ }$; (**b**) $5^{\circ }$; (**c**) $-5^{\circ }$; (**d**) $10^{\circ }$; (**e**) $-10^{\circ }$; (**f**) $15^{\circ }$; and (**g**) $-15^{\circ }$.

The comparison between electronic compass data and experimental data for the roll angle are presented in Figure 7 at different pitch angles. The red line indicates the compass data, and the blue line indicates the experimental data. The yellow histogram shows the error between the experimental data and the compass data. At each fixed pitch angle, the roll angle was varied from $-15^{\circ }$ to $15^{\circ }$ and measured every $5^{\circ }$ to obtain seven sets of roll angle values. For the whole range of pitch angle from $-15^{\circ }$ to $-40^{\circ }$, the experimentally determined roll angles are almost coincident with the actual angles at each measurement point on each graph, indicating the high accuracy of the experimental determinations. Similarly, Figure 8 compares the pitch angle measurement results at different roll angles. In this case, at each fixed roll angle, the pitch angle was varied from $-15^{\circ }$ to $-40^{\circ }$ and measured every $5^{\circ }$ to obtain six sets of pitch angle values. For the whole range of roll angles from $-15^{\circ }$ to $15^{\circ }$, the experimental pitch angle again coincided with the actual angle at each measurement point on each graph. Thus, overall, the method for attitude angle determination presented in this paper achieves high accuracy over a wide range of angles.

**Figure 9:**
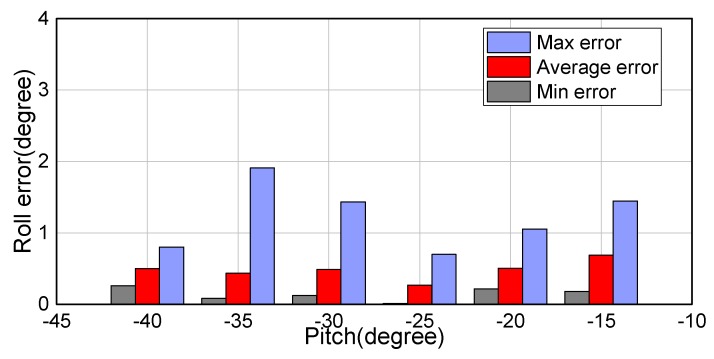
Errors in roll angle at different pitch angles.

**Figure 10:**
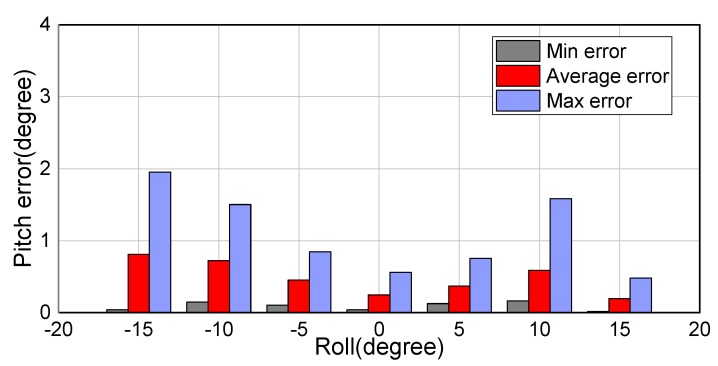
Errors in pitch angle at different roll angles.

An error analysis for the roll angle is presented in Figure 9, which shows the maximum, minimum, and average errors (errors here are absolute). It can be seen that the average error in the roll angle is $0.49^{\circ }$, $0.43^{\circ }$, $0.48^{\circ }$, $0.27^{\circ }$, $0.50^{\circ }$, and $0.68^{\circ }$ at the different fixed pitch angles. In particular, the low average error in the roll angle of $0.27^{\circ }$ at a pitch angle of $-25^{\circ }$ should be noted. The shooting angle of the camera also affects the accuracy of the drone’s attitude angles to a certain extent. Figure 10 presents a similar error analysis for the pitch angle. The average error in the pitch angle is $0.81^{\circ }$, $0.72^{\circ }$, $0.45^{\circ }$, $0.25^{\circ }$, $0.37^{\circ }$, $0.59^{\circ }$, and $0.19^{\circ }$ at the different fixed roll angles. It can be seen that when the roll angle changes from $0^{\circ }$ to $-15^{\circ }$, the average error in the pitch angle increases, reaching $0.81^{\circ }$ at a roll angle of $-15^{\circ }$. Furthermore, it can be seen from the plots in Figure 10 that when the actual value of the roll angle is $-15^{\circ }$, the experimentally determined values show clear deviations. Thus, it can be seen that increasing roll angle results in increasing errors in both pitch and roll angles.

The greatest errors in both pitch and roll angles (almost $2^{\circ }$) occur at a pitch angle of $-35^{\circ }$ and a roll angle of $-15^{\circ }$. From an analysis of the sources of these errors, it appears that the major contribution comes from image processing. Extraction of the center of the coded point is affected by the shooting angle. When this angle is skewed, the center after processing will deviate from the true center, resulting in an error in the extraction of the image center coordinates. This eventually leads to a deviation of the calculated result from the true value. In addition, the quality of the captured image also affects the accuracy of attitude angle determination.

### 4.4. Way Ahead

The proposed method in this paper is developed on the basis of visual measurement, which has some limitations at this stage and can be improved in the near future. The quality of the captured image is sensitive to the light condition, which directly depends on the weather condition. In some weather with poor light conditions, the proposed method may not be able to solve the attitude angle information due to the insufficient image quality. If precise and restricted landing direction and location are imposed as implemented, the navigation and control system are essential for the UAVs, which raise a new challenge for the treatment speed and the robustness of the proposed method. The accuracy of the attitude angle can also be improved by rationally designing the target size according to the working distance. It is worth mentioning that the size of the target depends on the camera focal length. Large focal length is required when shooting distance reaches hundreds of meters. The 200 mm focal length is able to capture a 4-m wide target 100 m away and a 2-m wide target 50 m away. In view of the limitation mentioned above, a series of research works are planned for the practical navigation including the optimization of target design, error model optimization, and flight control algorithm in the near future. In order to improve the robustness at poor light condition, self-illuminating targets that automatically adjust the brightness according to the light intensity will be explored, and the error model of attitude determination concerned with precise extraction of the cooperation center should be further studied to improve the accuracy of attitude determination.

The purpose of the method proposed in this paper is to achieve a precise landing of a small fixed-wing UAV under a high-precision attitude angle measurement system. At this stage, the attitude angle measurement system proposed in this paper is only used for the static measurement of the attitude angle, which simulates the variation of attitude angles at the landing stage. In the follow-up work, the measurement system will be applied to the practical small-scale fixed-wing UAV to achieve dynamic measurement of the attitude angle during landing, and the stability of the algorithm requires being further strengthened to ensure the real-time measurement of the attitude angle. Ultimately, the high-precision landing of the small-scale fixed-wing UAV will be achieved on the basis of the dynamic measurement of the attitude angle and a novel control algorithm and the autonomous control systems. It should be noted that the vision based measurement method proposed in this paper has the potential to develop the intelligence navigation—for example automatic obstacle avoidance, when combined with vision information and artificial intelligence technology. Furthermore, the novel attitude angle measurement is not only applicable for UAVs, but also possible for the application of other vehicles such as underwater vehicles, and these will also serve as future research projects for our group.

## 5. Conclusions

This paper proposes a method for attitude angle measurement using a single captured image to assist with the landing of small-scale fixed-wing UAVs. Compared with existing approaches, the proposed method has the advantage that the attitude angles are obtained from just one image containing five coded landmarks, which reduces the time to solve the attitude angle while having more than one image in most methods. In addition, this method can be adapted to use a zoom system that is able to improve the accuracy of the measured attitude angle while using the fixed focus length in most methods. Experimental results show a measurement accuracy of better than $1^{\circ }$ over wide attitude angle ranges, with pitch angle increasing from $-40^{\circ }$ to $-15^{\circ }$, roll angle decreasing from $+15^{\circ }$ to $-15^{\circ }$ and yaw angle decreasing from $+15^{\circ }$ to $-15^{\circ }$. Furthermore, the proposed method achieves an average error of $0.25^{\circ }$ at a roll angle of about $0^{\circ }$. The error in the attitude angles gradually increases as the roll angle departs from $0^{\circ }$. It is possible to achieve high accuracy during the whole UAV descent procedure, provided that the roll angle remains nearly constant. The results presented here indicate that the proposed method has great potential for assisting with the landing of small-scale fixed-wing UAVs. With the rapid development of artificial intelligence, the vision-based attitude angle measurement technology will have a broader application in the future.

## Figures and Tables

**Table 1:** Decoding table.

$X_{w}$ (mm)	$Y_{w}$ (mm)	Coded Value	*u* (Pixels)	*v* (Pixels)
40	110	5	199.5862	821.9177
180	110	7	802.5335	828.7201
110	180	10	511.8458	686.4126
40	250	13	243.7102	553.7738
180	250	15	801.7880	561.2718

**Table 2:** Attitude angle at different focal lengths.

Focal Length (mm)	Attitude Angle	Compass Data (Degree)	Experimental Data (Degree)	Error (Degree)
12	Pitch	−25.38	−24.90	−0.48
	Roll	−0.06	0.98	−1.04
	Yaw	−1.12	−1.22	−0.10
16	Pitch	−25.34	−24.47	−0.87
	Roll	−0.65	−0.95	0.30
	Yaw	−0.34	−0.38	0.04
25	Pitch	−25.10	−25.14	0.04
	Roll	−0.28	−0.53	0.25
	Yaw	−0.38	−0.32	−0.06
35	Pitch	−25.34	−25.38	0.04
	Roll	−0.67	−0.66	−0.01
	Yaw	0.88	2.20	−1.32

**Table 3:** Comparison of yaw angles with roll angles changed from $-15^{\circ }$ to $15^{\circ }$.

Roll	–15	–10	–5	0	5	10	15
**Compass data (degree)**	−0.29	−0.23	−0.49	−0.38	0.70	−0.37	−0.33
**Experimental data (degree)**	0.17	0.21	−0.76	−0.33	1.01	0.56	−0.89
**Error (degree)**	−0.46	−0.44	0.27	−0.05	−0.31	−0.93	0.56

**Table 4:** Comparison of yaw angles with pitch angles changed from $-15^{\circ }$ to $-40^{\circ }$.

Pitch	–15	–20	–25	–30	–35	–40
**Compass data (degree)**	−1.02	−0.80	−0.78	−0.38	−0.14	−0.30
**Experimental data (degree)**	−1.64	−1.37	−1.57	−0.33	−0.46	−0.91
**Error (degree)**	0.62	0.57	0.79	−0.05	0.32	0.61

**Table 5:** Results of attitude determinations at six different positions.

Position	Attitude Angle	Compass Data (Degree)	Experimental Data (Degree)	Error (Degree)
One	Pitch	−15.23	−15.35	0.12
	Roll	0.33	1.06	−0.73
Two	Pitch	−20.79	−21.35	0.56
	Roll	−0.24	0.52	−0.76
Three	Pitch	−25.34	−25.38	0.04
	Roll	−0.67	−0.66	−0.01
Four	Pitch	−30.05	−29.96	−0.09
	Roll	−0.18	−0.10	−0.08
Five	Pitch	−35.42	−35.78	0.36
	Roll	−0.21	−0.20	−0.01
Six	Pitch	−40.34	−40.04	−0.30
	Roll	−0.12	0.18	−0.30
